# Supplementary material for: Influencing factors of HCV testing willingness among men who have sex with men in China: A structural equation modeling analysis
Source: PLoS One. 2025 Apr 3;20(4):e0321469. doi: 10.1371/journal.pone.0321469 (PMC11967942; doi:10.1371/journal.pone.0321469)
Supplement: Attachment — Submitted filename: Response to reviewers.docx [file pone.0321469.s003.docx]

Dear Editor and Reviewers:

Thank you for your letter and for the reviewers’ comments concerning our manuscript entitled “Influencing Factors of HCV testing Willingness among Men Who Have Sex with Men in China: A Structural Equation Modeling Analysis” (ID: PONE-D-24-46278). We wish to thank you all for your constructive comments. Your comments provided valuable insights to refine its contents and analysis. In this document, we try to address the issues raised as best as possible.

We have incorporated most of the suggestions made by the reviewers. Those changes are highlighted within the manuscript. Please see below, in blue, for a point-by-point response to the reviewers’ comments and concerns. All page numbers refer to the revised manuscript file, accompanied by tracked changes.

Once again, we thank you for the time you put in reviewing our paper and look forward to meeting your expectations.

Yours sincerely,
Jianyan Li

Corresponding author: 
Name: Xiaoni Zhong
E-mail: zhongxiaoni@cqmu.edu.cn

**Journal Requirements:**

1. Please ensure that your manuscript meets PLOS ONE's style requirements, including those for file naming. The PLOS ONE style templates can be found at https://journals.plos.org/plosone/s/file?id=wjVg/PLOSOne_formatting_sample_main_body.pdf and https://journals.plos.org/plosone/s/file?id=ba62/PLOSOne_formatting_sample_title_authors_affiliations.pdf

Response: Thank you for pointing this out. We have revised the manuscript and the file name to meet the style requirements of PLOS ONE.

2. Please provide additional details regarding participant consent. In the ethics statement in the Methods and online submission information, please ensure that you have specified (1) whether consent was informed and (2) what type you obtained (for instance, written or verbal, and if verbal, how it was documented and witnessed). If your study included minors, state whether you obtained consent from parents or guardians. If the need for consent was waived by the ethics committee, please include this information.

If you are reporting a retrospective study of medical records or archived samples, please ensure that you have discussed whether all data were fully anonymized before you accessed them and/or whether the IRB or ethics committee waived the requirement for informed consent. If patients provided informed written consent to have data from their medical records used in research, please include this information.

Response: We have included the following information in the ethics statement in both the Methods and online submission information. The participants were informed consent. Before starting the survey, the study objectives were informed to the participants. Respondents were adequately informed that their information would be confidential and no identifiable information would be disclosed. Moreover, it is clearly stated that the participants can withdraw from the survey at any point. We obtained implied consent. We have provided a detailed description of the above content on the first page of the questionnaire. If participants agree to take part in the study, they can proceed to the next page on their own to fill out the questionnaire. Since it is implied consent, there is no written or oral consent in the traditional sense. In addition, our research does not include minors.

3. Thank you for stating the following financial disclosure:

This work was supported by the National Key Project for Infectious Diseases of the

Ministry of Science and Technology of China (2018ZX10721102-005).

Please state what role the funders took in the study. If the funders had no role, please state: ""The funders had no role in study design, data collection and analysis, decision to publish, or preparation of the manuscript.""

If this statement is not correct you must amend it as needed.

Please include this amended Role of Funder statement in your cover letter; we will change the online submission form on your behalf.

Response: We have stated in the manuscript that the funders had no role in study design, data collection and analysis, decision to publish, or preparation of the manuscript.

4. Ethics statement only appears at the end of the manuscript:

Your ethics statement should only appear in the Methods section of your manuscript. If your ethics statement is written in any section besides the Methods, please move it to the Methods section and delete it from any other section. Please ensure that your ethics statement is included in your manuscript, as the ethics statement entered into the online submission form will not be published alongside your manuscript.

Response: Thank you for pointing this out. We have moved the ethical statement to the Methods section and deleted it from other parts.

5. We note that you have indicated that there are restrictions to data sharing for this study. For studies involving human research participant data or other sensitive data, we encourage authors to share de-identified or anonymized data. However, when data cannot be publicly shared for ethical reasons, we allow authors to make their data sets available upon request. For information on unacceptable data access restrictions, please see http://journals.plos.org/plosone/s/data-availability#loc-unacceptable-data-access-restrictions.

Before we proceed with your manuscript, please address the following prompts:

If there are ethical or legal restrictions on sharing a de-identified data set, please explain them in detail (e.g., data contain potentially identifying or sensitive patient information, data are owned by a third-party organization, etc.) and who has imposed them (e.g., a Research Ethics Committee or Institutional Review Board, etc.). Please also provide contact information for a data access committee, ethics committee, or other institutional body to which data requests may be sent.

If there are no restrictions, please upload the minimal anonymized data set necessary to replicate your study findings to a stable, public repository and provide us with the relevant URLs, DOIs, or accession numbers. Please see http://www.bmj.com/content/340/bmj.c181.long for guidelines on how to de-identify and prepare clinical data for publication. For a list of recommended repositories, please see https://journals.plos.org/plosone/s/recommended-repositories. You also have the option of uploading the data as Supporting Information files, but we would recommend depositing data directly to a data repository if possible.

Please update your Data Availability statement in the submission form accordingly.

Response: We understand the strict requirements of the journal for data sharing and transparency. At the same time, we always regard the protection of participants' privacy as of utmost importance. Previously, due to concerns about the privacy information of the participants, we chose not to share the de-identified data. However, in order to actively cooperate with the requirements of the journal, we have taken further measures to remove all parts of the data that can identify personal information, ensuring that there is no risk to the privacy of the participants. In view of the fact that we have now completed the data de-identification process, we have uploaded the processed data set as a "supporting information" file, so that you and other interested researchers can obtain the data for further verification and research.

6. Please include a separate caption for each figure in your manuscript.

Response: Thank you for pointing this out. We have added a separate caption to each figure. We uploaded the figures as individual files.

7. Please include your tables as part of your main manuscript and remove the individual files. Please note that supplementary tables (should remain/ be uploaded) as separate ""supporting information"" files.

Response: Thank you for pointing this out. We have moved the tables from the end of the manuscript to their corresponding positions within the manuscript.

Thank you again for your constructive comments and suggestions on our manuscript.

**Reviewer 1 Comments**

Points in general:

Please make sure to use fair, accurate and respectful language and use people first language (See NIAID HIV Language Guide and People First Charter) throughout the manuscript. Some of the words or combination of words the authors use can be stigmatizing. This is very important.

Response: We sincerely appreciate your pointing out the issues regarding the language use in our paper. This is of great significance for us to improve the paper. We carefully referred to the NIAID HIV Language Guide and the People First Charter, examined the entire paper, and made revisions as required. We currently believe that the language in the paper adheres to the principles of fairness, accuracy, and respect. However, if you could specify the words or combinations that are considered stigmatizing, we will make the modifications immediately to ensure the rigor and appropriateness of the language in the paper.
Thank you again for your valuable comments.

1. Abstract:

- Line 23: I am puzzled by the use of detection rate, and wonder whether the authors actually mean testing rate? Detection rate relates to the number of positive HCV tests among those that were tested for HCV. Given your aim to assess influencing factors of HCV testing willingness, you might want to use testing rate instead of detection rate.

Response: We are very sorry for our negligence. We have changed "detection rate" to "testing rate".Please see lines 23-25 in the manuscript.

2. Introduction:

- Line 65-66: Do the authors mean anti-HCV antibody prevalence or RNA prevalence?

Response: Thank you for pointing this out. We read the literature again, but the author of this reference mentioned the prevalence without specifying whether it is the anti-HCV antibody prevalence or the RNA prevalence.

The original text is as follows：“We did a systematic review and meta-analysis of studies published and listed on MEDLINE or Embase between Jan 1, 2000, and Oct 31, 2019, including conference proceedings. Studies were eligible if they reported measures of HCV prevalence or HCV incidence (or both) among MSM. Studies that relied on participants' self-reported HCV status with no laboratory confirmation were excluded.”“Overall, the pooled HCV prevalence in MSM was 3.4% (95% CI 2.8–4.0; *I*^2^=98.0%) .” “ Globally, HCV prevalence was 1.5% (1.0–2.1) in HIV-negative MSM and 6.3% (5.3–7.5) in HIV-positive MSM. ”

- Line 69: Had ever or recently undergone HCV testing? Please be more specific here.

Response: We are extremely grateful to Reviewer for pointing out this problem. We've made the modification, the revised sentence is as follows (Lines 71 in the manuscript): just 41% of Chinese MSM had ever undergone HCV testing.

- Also see my previous comment related to the background section: I am questioned whether you actually mean detection rate or testing rate? The 41% you are referring to is a testing rate.

Response: We are very sorry for our negligence of these problems. What we meant was the testing rate. We've made the modification, please refer to lines 69 - 70 of the manuscript.

3. Methods:

- Line 84-89: The in- and exclusion criteria are not entirely clear to me. Why do the authors exclude those with a significant mental disease or intellectual disability, and how do the authors determine that? And why exclude those who took fewer than three minutes to complete the questionnaire?

Response: Thank you for pointing this out. We do this to avoid situations where individuals are unable to fill out the questionnaire properly due to mental illness or intellectual disabilities. We determine this by checking whether they can fill out the questionnaire normally. To make the expression clearer, we have made revisions to the original sentence.The new sentence reads as follows: (2) be able to fill out the questionnaire independently. (Line 90 in the manuscript)

After testing, we found that it takes at least 3 minutes to complete this questionnaire when the questions are fully understood. Therefore, to avoid the situation of respondents answering without fully understanding the questions, we excluded the responses completed in less than 3 minutes. We have made revisions to make the expression clearer. Please see lines 92-93.

- Line 96-99: This is part of the results section, and is preferably mentioned at the very start of the results section.

Response: Thanks for your constructive suggestion. We have moved this part to the beginning of the Results section as reviewer's suggestion. Please see lines 172-175 in the manuscript.

- Line 114-116: It would be very helpful if the authors elaborate a bit more on the 23-item scale. Initially, it is not clear what the 23-item scale helps to assess and what it is for. And as the authors mention that the scale was based on the HBM and previous research, I also miss a reference. In the discussion part, the authors explain the definitions of the categories, but I would suggest to mention that in the Method section as this information is critical to understand the work.

Response: Thanks for your kind suggestions.We have added the purpose of the scale according to the reviewer's suggestions. Please see lines 120-121 in the manuscript.

In addition, we have supplemented the references of previous similar studies. Please see line 120.

Finally, we have also placed the definition of the categories in the Methods section. Please see lines 125-137.

- Could you please elaborate a bit more on the reference periods you use in the questionnaire? Is it ever tested for HCV, or in the past six months? And also do this for other variables. This is important to understand your results and translate them to current practice.

Response: We are very sorry for our negligence of these problems. We've added the reference period for this variables. Please see table 2.

- What are the current HCV testing recommendation among MSM in China? This information would be much needed to understand the results of this work and to translate it to any implications. Is a testing rate of 55% really that low?

Response: Thank you for pointing this out. The "Work Plan for Actions to Eliminate the Public Health Hazards of Hepatitis C (2021 - 2030)" in China emphasizes the need to intensify HCV testing efforts, increase the detection rate, implement the strategy of "testing all who should be tested" in medical institutions and among key populations, and the strategy of "testing all who are willing to be tested" among the general public.

The MSM population has relatively high risk factors for HCV infection. A 55% testing rate reflects a certain level of awareness of detection. However, based on the above work plan, we believe there may still be room for improvement in this figure.

In addition, regardless of whether the 55% testing rate is high or low, this value indicates that we need to strengthen HCV test among the MSM population. This is because the rate of chronic HCV infection is relatively high. Early detection and treatment are crucial for preventing disease progression and reducing the occurrence of serious consequences such as liver cirrhosis and liver cancer. Even if the testing rate is relatively high, nearly half of the high - risk population may not have been tested. This part of the population is at potential risk of infection and may become hidden sources of disease transmission, further expanding the scope of transmission. Therefore, we should take more measures to increase the testing rate.

- And I wonder whether the authors also collected data on HIV status, as nothing is mentioned about that. We know from previous literature that new HCV infections are mostly found among MSM with HIV and MSM using HIV PrEP. Ofcourse, the epidemiological context of HCV might be different in China compared to Europe and the Amercas, but since MSM are considered a key population in China as well, this might also be the case there.

Response: Thank you for pointing this out. In this study, we only collected data on HIV testing among MSM, without gathering the specific test results. The findings show that 65.8% of them had ever undergone HIV testing. The reason for not collecting data on HIV status is mainly that the core of this research focused on the influencing factors of the willingness to test for HCV. At that time, the research design did not include the HIV infection status as a major variable.

However, we are fully aware of the close connection between HIV infection status and HCV infection. As you mentioned, previous literature indicates that among the MSM, HIV - positive individuals and those using HIV PrEP are indeed high - risk groups for new HCV infections. In subsequent research plans, we will take the collection of HIV infection status data into important consideration. We hope that by supplementing this information, we can further explore in - depth the co - infection patterns of HIV and HCV among MSM, providing a strong basis for formulating more targeted prevention and control strategies.

Once again, thank you for your professional advice.

- The Method section could be extended a bit more. I am missing some information about the variables that were collected in the study, and the definitions that were used. For instance, looking at the method sectoin, it remains unclear that the authors collected data on STD and what type of STDs were included in this definition (all type of STDs, or only chalmydia, gonorrhea and syphilis?). I would suggest to mention this in the method section. For instance: We collected self-reported data on whether participants were diagnosed with any sexually transmitted infection, including XX, XX and XX. I also miss the reference period, is it ever diagnosed with an STD, or diagnosed in the past six months?

Response: Thanks for your constructive suggestion. We have improved the methods section according to the Reviewer’s suggestion. We have listed most of the collected variables, all of which are marked with the reference period. In addition, Sexually transmitted diseases refer to all types of sexually transmitted diseases that have ever been diagnosed. Please see lines 103-109 and table 2.

4. Results

- Line 155: Had been tested for HCV in the past six month, or ever? Again, reference periods are unclear.

Response: We are very sorry for our negligence. We've added the reference period for this variables. The new sentence reads as follows: 55.7% had been ever tested for HCV. (Lines 182-183 in the manuscript)

- Line 155-156: Willing to be tested for HCV in the future, or in the upcoming six month? Reference periods are unclear.

Response: We are very sorry for our negligence. We've added the reference period for this variables. The new sentence reads as follows: 90.9% of MSM said they were willing or very willing to be tested for HCV in the future, and 71.7% of MSM said they were high or very likely to be tested for HCV in the next six months. (Lines 183-185 in the manuscript)

5. Discussion

- Line 185: Here the authors talk about testing rate, make sure to be consistent throughout the paper using testing rate.

Response: We are very sorry for our negligence of these problems. We have made corresponding revisions to ensure consistency throughout the article.

- Line 189-190: Higher willingness to test for HCV compared to? Or just a high willingness to test for HCV?

Response: We are very sorry for our negligence. We have made corresponding modifications. The new sentence reads as follows: MSM have a high willingness to test for HCV. (Line 230 in the manuscript)

- In the discussion, the authors explain what is meant with self-efficacy, perceived susceptibility, perceived severity, perceived advantage. This type of information belongs to the method section, as it helps to understand the results.

Response: Thanks for your kind suggestions. We have moved these explanations to the methods section. Please see lines 125-137.

- 55% of the sampled population reported being tested for HCV. Is that really that bad when almost two thirds of the sampled population only had one sexual partner, and thus might not be susceptible for HCV acquisition? I agree with the author’s limitation that the results may not be generalizable to other groups of MSM, but 55% might not even be that bad? But again, I don’t know the reference period of the question and the current HCV testing guidelines in China.

Response: We are extremely grateful to Reviewer for pointing out this problem. We have added the reference period for the question. It asked whether the participants had ever been tested for HCV. (Line 226 and table 2). In addition, the "Work Plan for Actions to Eliminate the Public Health Hazards of Hepatitis C (2021 - 2030)" in China emphasizes the need to intensify HCV testing efforts, increase the detection rate, implement the strategy of "testing all who should be tested" in medical institutions and among key populations, and the strategy of "testing all who are willing to be tested" among the general public.

Although approximately two - thirds of the sampled population reported having only one sexual partner, this does not mean they are less likely to be infected with HCV. The transmission routes of HCV are diverse. It not only includes sexual transmission but can also occur through blood - borne transmission. For example, infections may happen during daily activities that can lead to blood exposure, such as tattooing, piercing, sharing toothbrushes or razors. Even in terms of sexual transmission, having only one sexual partner does not necessarily guarantee complete safety. If this partner engages in other high - risk behaviors (such as injecting drug use, having sex with other individuals who may be infected with HCV, etc.), the risk of infection still exists.

In addition, 55% of the sampled population had been tested for HCV in the past. Although this appears to be a certain proportion, it still implies that a significant portion of the high - risk population have never been tested. Given that the process of HCV infection becoming chronic can be latent, the undetected infected individuals may unconsciously progress to chronic hepatitis, which could then trigger serious consequences such as liver cirrhosis and liver cancer. Moreover, they may continue to spread the virus unawares, posing a potential threat to public health. Therefore, even considering the fact that some people have only one sexual partner, we believe that there is still room to increase this testing rate. And enhancing the testing rate of this population is of vital importance for controlling the spread of HCV and the progression of the disease.

Thank you again for your question. It helps us think more comprehensively about the issues in the research.

Thank you again for your constructive comments and suggestions on our manuscript.

**Reviewer 2 Comments**

1.The author could add specific objectives of the study.

Response: Thanks for your constructive suggestion. We have supplemented this section according to the Reviewer’s suggestion. Please see lines 76-82.

2.In methodology the author could briefly explain the process of authentication of the recruited participants as it was an online recruitment. Author can clarify were the participants aware of the reward of 5 RMB (about $0.69) before giving consent or not. If yes, it can create ethical issues, which can be explained.

Response: Thanks for your constructive suggestion. During the online recruitment process of this study, we adopted multi - dimensional approaches to ensure the accuracy and reliability of participant identity verification. In terms of recruitment channels, MSM volunteers were sought via the non-probability sampling method, making use of online platforms such as WeChat official accounts frequently used by MSM, peer introductions, non-governmental organization (NGO) collaboration, and the "snowballing" of core members. These channels can precisely reach the target population and guarantee the pertinence of the source of participants. Regarding the identity confirmation section, we set relevant questions in the questionnaire. These questions include "Gender" and "Have you had sexual relations with men in the past year?" to further determine whether the participants belong to the MSM population. Only those participants who meet the criteria will be included in the study. In addition, we configured the questionnaire such that only one submission is allowed for the same device, IP address, and account, thus ensuring the uniqueness of responses. We briefly explained the authentication process for recruited participants. Please see lines 86-87 and 96.

We have explained in the manuscript that participants were informed of the 5 - yuan reward before they gave their informed consent. Please see lines 99-100. During the entire recruitment and informed consent process of the study, we strictly adhered to the relevant requirements of ethical review. While introducing in detail to the participants information such as the purpose, process, risks, and benefits of the study, we clearly informed them that they would receive a certain amount of compensation for their participation, including the specific amount of the compensation (i.e., 5 yuan). This approach aims to ensure that participants can fully understand all aspects of their participation in the study, including the material incentives, so that they can make an autonomous and informed decision. The 5 - yuan RMB compensation was determined based on comprehensive factors such as the local economic level, the nature of the study, and the time cost. The purpose is to provide a reasonable reimbursement for the time and effort they spent participating in this study, without exerting excessive influence on the participants' decisions. Additionally, according to previous studies, responses to the questionnaire can be increased by giving incentives. As Edwards, P. J., Roberts, I., Clarke, M. J., DiGuiseppi, C., Woolf, B., and Perkins, C. (2023) stated in their work "Methods to increase response to postal and electronic questionnaires": "Response to questionnaires can be increased by giving an incentive, for example, a small amount of money, or a non‐monetary incentive such as a pen".

Thank you again for your suggestions. We will continue to maintain a high level of ethical awareness in future research to ensure the scientific and ethical nature of the research process.

3.Likewise, author could briefly explain the meaning of problems in logical check and the way to identify it.

Response: Thanks for your kind suggestions. We have made a brief explanation in the corresponding section.The new sentence reads as follows:

The logical test questions were answered incorrectly, or the answers to some questions in the questionnaire were contradictory. (Lines 94-95 in the manuscript)

In this study, the questionnaire contains logical test questions such as “3 + 2 =?”. In addition, logical screening includes the following: marital status is married but under the legal age of marriage; having had sex in the past six months but having chosen zero sexual partners; having had group sex in the past six months but choosing less than or equal to one sexual partner; the answers of the questionnaire were highly consistent and so on.

4.In the Table 2 legend: Characteristics of the study sample (N=857), however in the table N(n=680), which is not matching, and clarified by the author.

Response: We are very sorry for our negligence of these problems.This was a writing mistake made by the author and has been rectified.

5.Conclusion could be aligned to aim & objectives of the study, the author could suggest recommendations according to the study results.

Response: Thanks for your constructive suggestion. In accordance with the reviewer's comments, the conclusion section has been revised to align with the research objectives and purposes. Additionally, recommendations have been put forward based on the research findings. Please see lines 294-300.

Thank you again for your constructive comments and suggestions on our manuscript.
